# P-128. Evaluating dopamine agonists to confer colonization resistance against bacterial enteric infection: population-based propensity-matched cohort study

**DOI:** 10.1093/ofid/ofae631.333

**Published:** 2025-01-29

**Authors:** Morgan Birabaharan, David C Kaelber, Sanjay R Mehta

**Affiliations:** UC San Diego School of Medicine, San Diego, California; MetroHealth Medical Center/ Case Western Reserve University, Cleveland, Ohio; San Diego VA Medical Center and University of California San Diego, San Diego, California

## Abstract

**Background:**

Uncovering gut microbiota mechanisms can afford new preventive therapies against enteric infection amidst the antimicrobial resistance crisis. Recent groundbreaking work in mice models suggest microbial-mediated metabolism of diet-derived tryptophan helps confer colonization resistance against enteric pathogens via agonism of dopamine receptors. This study aims to assess the risk of bacterial enteric infection in restless leg syndrome patients using a dopamine agonist (ropinirole) compared to a voltage-gated calcium channel modulator (gabapentin).

**Table 1:**
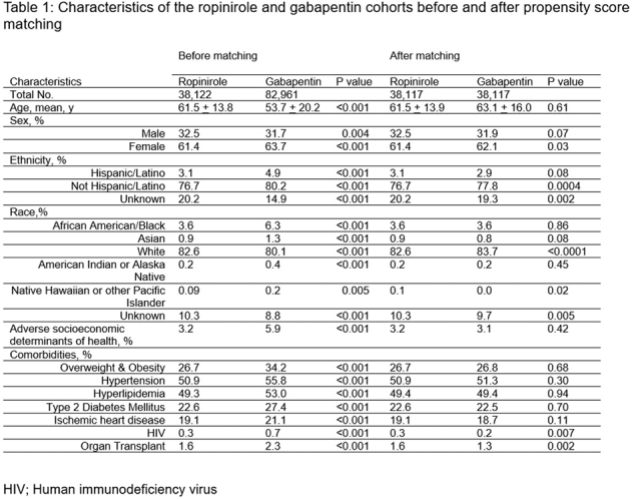
Characteristics of the ropinirole and gabapentin cohorts before and after propensity score matching

**Methods:**

The TriNetX Analytics Platform containing 89 million unique patients from 63 health care organizations was used. These data represent 27% of the US population from all 50 states, and includes persons from diverse geographic, age, race, ethnicity, income, and insurance groups. Persons >18 years of age diagnosed with restless leg syndrome and prescribed ropinirole or gabapentin between Jan 2018- Jan 2023 were included as the study and control cohort, respectively. Individuals who received both ropinirole and gabapentin were excluded. The two cohorts were propensity score matched for age, sex, race, ethnicity, social determinants of health, obesity, ischemic heart disease, type 2 diabetes mellitus, hypertension, hyperlipidemia, human immunodeficiency virus and organ transplant status. Using the matched cohorts, we calculated the relative risk of first-ever and recurrent bacterial enteric infection 1-year after index events in the respective cohorts.

**Results:**

We identified 38,122 and 82,961 persons with restless leg syndrome receiving ropinirole or gabapentin, respectively. After 1:1 propensity matching, demographic and clinical characteristics were balanced (SD < 0.1) (Table 1). Ropinirole compared to gabapentin use among persons with restless leg syndrome was associated with decreased 1-year risk of both recurrent (RR 0.85, 95% CI 0.74-0.98) and new-onset bacterial enteric infection (RR 0.77, 95% CI 0.65-0.92).

**Conclusion:**

This study found that persons with restless leg syndrome receiving dopamine-agonist (ropinirole) compared to voltage-gated calcium channel modulator therapy (gabapentin) was associated with reduced risk of recurrent and new-onset bacterial enteric infection.

**Disclosures:**

**David C. Kaelber, MD, PhD, MPH, FAAP, FACP, FACMI, FAMIA**, Dynavax Technologies Corporation: Advisor/Consultant

